# The “Bad Father”: Paternal Role in Biology of Pregnancy and in Birth Outcome

**DOI:** 10.3390/biology13030165

**Published:** 2024-03-03

**Authors:** Stefano Raffaele Giannubilo, Daniela Marzioni, Giovanni Tossetta, Ramona Montironi, Maria Liberata Meccariello, Andrea Ciavattini

**Affiliations:** 1 Clinic of Obstetrics and Gynaecology, Department of Clinical Sciences, Università Politecnica Delle Marche, 60123 Ancona, Italy; ramonamontironi@gmail.com (R.M.); liberamec@hotmail.it (M.L.M.); a.ciavattini@staff.univpm.it (A.C.); 2 Department of Experimental and Clinical Medicine, Università Politecnica Delle Marche, 60126 Ancona, Italy; d.marzioni@staff.univpm.it (D.M.); g.tossetta@pm.univpm.it (G.T.)

**Keywords:** father, paternal, placenta, pre-eclampsia, pregnancy, preterm

## Abstract

**Simple Summary:**

Human reproduction, as well as that of all mammals, involves the union of two cells, the male sperm and the female egg, which give rise to a new organism that will grow for about 280 days inside the mother’s body. Most research on pregnancy, its complications, and diseases of the unborn child and newborn has focused, appropriately enough, on maternal conditions and the interaction between mother and child, leaving the father with only the role of depositing his genetic material at the moment of conception. This study aims to compile the research that has dealt with the father’s role in determining a good or bad course of pregnancy and birth. From this perspective, the father can be a “good father” or a “bad father” not only because of his hereditary genetic heritage, but also because of how he lives, how he feeds, and how he eats; in short, if a man takes care of his health, he is already taking care of his children’s health.

**Abstract:**

Pregnancy is generally studied as a biological interaction between a mother and a fetus; however, the father, with his characteristics, lifestyle, genetics, and living environment, is by no means unrelated to the outcome of pregnancy. The half of the fetal genetic heritage of paternal derivation can be decisive in cases of inherited chromosomal disorders, and can be the result of de novo genetic alterations. In addition to the strictly pathological aspects, paternal genetics may transmit thrombophilic traits that affect the implantation and vascular construction of the feto-placental unit, lead to placenta-mediated diseases such as pre-eclampsia and fetal growth retardation, and contribute to the multifactorial genesis of preterm delivery. Biological aspects of immunological tolerance to paternal antigens also appear to be crucial for these pathologies. Finally, this review describes the biological findings by which the environment, exposure to pathogens, lifestyle, and nutritional style of the father affect fetal pathophysiological and epigenetic definition.

## 1. The Father and Pregnancy Loss

Recurrent pregnancy loss (RPL) is defined as three or more consecutive abortions and constitutes about 1% of all cases of pregnancy loss [1]. RPL is a multifactorial disease and recognizes several causes, including anatomic (uterine malformations), endocrine, infectious, immunologic, genetic, and idiopathic. The genetic causes are estimated to affect 50% of abortions, and there are still not enough studies on the role of paternal factors in RPL [2]. The most common structural abnormality which can impact reproductive success is balanced translocation, a translocation or reciprocal inversion (or Robertsonian), with a prevalence of 0.1% in the population [3]. However, in couples with repeat abortions, the prevalence reaches the 8% [4]. For example, if a dad is a carrier of a 21q21q translocation, the risk of generating trisomy 21 will be 100%, which can lead to an early abortion in 50–80% of cases [5]. When one of the parents is a carrier of a balanced translocation, the risk of Down syndrome increases; specifically, the risk would be 10–15% when the carrier is the mother and 2–3% when the carrier is the father [6]. In this regard, a higher incidence of disomy of the sex chromosomes [7] and a higher susceptibility of chromatin to acid denaturation [8] was detected in the spermatozoa of couples with repeated miscarriage and infertility. Regarding point mutations, the role of mutation of the gene for Human leukocyte antigen-G (HLA-G), a leukocyte antigen expressed from fetal tissues and the maternal–fetal interface was considered. A variant in the Alpha-2 domain of this gene is associated with repeat abortions (up to five abortions) [9]. Although there are contradictory reports available, with regard to the association of maternal thrombophilia with adverse pregnancy outcomes, there is extensive literature about the role of certain maternal thrombophilic mutations, including Leiden Factor V (rs6025), of the gene for prothrombin (***20210G > A***) (rs1799963), of methylenetetrahydrofolic reductase (***MTHFR 677C > T***) (rs1801133) in recurrent abortion [10,11]. This appears to be little studied on the paternal side, although extraembryonic tissues are composed of proteins encoded by genes from both parents. There is evidence that paternal hyperhomocysteinemia (from the mutation of the MTHFR gene) would increase the risk of miscarriage 6.92-fold [12]. The exact contribution of possible “fetal thrombophilia” inherited from the father has not been established [13,14]. Another paternal factor that may affect reproductive success is the microdeletion of the chromosome Y. Men with this defect and severe oligozoospermia or azoospermia are able to reproduce via assisted reproductive technologies. Although inheritance of the Y microdeletion apparently has no somatic effect on male offspring [15], there remains a risk that transmission of the microdeletions from father to son confers adverse effects on male fertility. Paternal age affects reproductive success on several fronts, from the increased prevalence of aneuploidies to reduced quality of spermatozoa (particularly motility), reduced reproductive rate, and the increased incidence of at least 20 autosomal dominant diseases (Apert syndrome, achondroplasia, Marfan syndrome, etc.). Paternal age is a known risk factor in cases of miscarriage, i.e., advanced age in men can increase the risk of spontaneous abortion [16]. The fact that seminal fluid can modulate both sperm–egg interaction and maternal reproductive apparatus responses [17] indicates the important role of seminal fluid in regulating reproduction. In general, events of chromosome non-disjunction with aging are more likely to occur, particularly in sex chromosomes, and, although most constitutional aneuploidies come from the female germ line, all men produce about 3–5% aneuploid sperm [18,19,20]. In addition, sperm quality and paternal obesity are related to fertilization rate and embryo development. Male obesity has been shown to compromise time to conception, fertilization rates, sperm capacitation, and the ability for the sperm to bind to the oocyte [21].

## 2. The Father and the Duration of Pregnancy

According to a Swedish study, it is the paternal genes that determine the duration of pregnancy. Olesen et al. [22], from the University of Aarhus, studied two groups of women who have had at least two children. The first group consisted of nearly 11,000 women whose first pregnancy had been prolonged (over 42 weeks), and the second group of 3500 women whose first pregnancy had ended within 42 weeks. The data reported in this study indicated that the risk that the second pregnancy would be prolonged was 19.9% in the first group, whereas it was 7.7% in the second group (regular pregnancy). However, if the two children were from different fathers, the probability that the second pregnancy would be prolonged decreased by 15.4%. In addition, compared with children born to the same father, those born from different fathers differed in the duration of pregnancies by more than one week. The study cited has epidemiological features and does not imply genetic significance. It remains that multiple biological, genetic, environmental, and behavioral (such as maternal stress) factors intervene in pregnancy duration; however, the suggestion remains that the phenomenology of pregnancy duration definitely passes through the maternal pathway but is under the influence of the father.

## 3. The Father and the Thrombophilic Fetus

Several studies have attempted to identify a correlation between adverse obstetric outcomes and maternal thrombophilia, with controversial results; this is particularly true when the expected fetal outcomes are similar to the maternal outcomes [23]. Several obstetrical pathologies have been studied and correlated with alterations in various thrombophilic factors, but some studies report conflicting results. Unexplained fetal death was most associated with heterozygosity of Factor V Leiden, protein C, protein S deficiency, and increased APCR. Intrauterine growth restriction (IUGR), finally, showed a higher prevalence of heterozygosity for Factor II, homozygosity for the ***MTHFR C677T*** mutation, and protein S deficiency [24]. Of particular interest is the role of the fetus and its possible thrombophilia even if the exact fetal contribution to placental disfunction is not yet well delineated. The hypothesis of a paternal role in the link between thrombophilia and obstetrical diseases stems from observations that the fetus may have an inherited thrombophilic state, such as the factor V Leiden mutation, not present in the mother [25,26]. The hypothesis contemplated concerns the delicate balance between pro- and anticoagulant factors at the feto-placental interface, therefore the phenomena of fibrin deposition and infarction could arise either from the maternal or fetal side, or from both. The expression of certain paternally derived allelic variants in the trophoblast involved in immune regulation or vascular remodeling has also been linked to adverse pregnancy outcomes associated with abnormal placentation [27,28]. The presence of microthrombi in the chorio-decidual capillaries could, in fact, compromise the physiological anchoring of the villi and lead to the phenomena ranging from activating the coagulatory “cascade” to the thrombosis of vessels in the umbilical cord or multiple placental infarcts. The double homozygosity for thrombophilic mutations necessarily invokes the presence of mutations in both paternal and maternal genetics. The study of placental tissue, in fact, leads to a shift in attention to “fetal thrombophilia” rather than maternal; thus the fetus, with the inherited genetic material, becomes the architect of its own destiny [29]. Hypercoagulable states at this level depend only on the fetal genotype, which is inherited from both parents [30]. This gives rise to the thrombophilic fetus hypothesis [31], since the thrombophilic-related fetal outcomes depend not only on the presence of maternal thrombophilia, but may be an expression of a fetal thrombophilic state inherited by the mother or the father or both. In the scenario of maternal thrombophilia and adverse fetal events, some authors [32] confirm this association for IUGR by asserting that the clinical variability in the severity of IUGR depends on the extent of maternal and/or fetal thrombophilic disorders. The picture instead becomes predictably clear when dealing with thrombotic feto-neonatal outcomes in fetuses (or infants) with thrombophilic factors. This association is confirmed and considered strongly in several case reports with perinatal stroke as their subject [33].

## 4. The Father and Pre-Eclampsia

Pre-eclampsia is a severe obstetric condition that complicates approximately 3–5% of pregnancies, being the major cause of maternal mortality in developed countries and one of the major causes of iatrogenic prematurity and low-birth-weight infants [34]. The basic pathophysiology of pre-eclampsia involves the incomplete invasion of maternal uterine spiral arteries by extravillous trophoblast cells [35,36]. A previous abortion participates in this regard, while the protective effect of a previous pregnancy is lost in the case of a change of partner [37]. In multiparous women with pre-eclampsia/HELLP syndrome (hemolysis, elevated liver enzyme level, low platelet count), a change of partner is more frequent than in physiological multiparas (25% vs. 3.4%) [38], and in pre-eclamptic women, there is a higher index of cohabitation and sexual intercourse with the partner of the current pregnancy. This, in fact, agrees with the observation of a higher incidence of pre-eclampsia in pregnant adolescents. In a study of a large Norwegian population, pregnancies were studied in all combinations of couples: same or different father and same or different mother in two successive pregnancies. It verified that a father who has “fathered” a pre-eclamptic pregnancy has a double risk of generating a pre-eclamptic pregnancy with another woman as well. This phenomenon has been referred to as “the dangerous father” [39]. The mammalian fetus, after the phase of implantation of the blastocyst, is intimately connected with the uterine tissues and the maternal blood system. From an immunological point of view, this is a real paradox: fetal tissues expressing antigens of paternal inheritance provoke the responses of the immune maternal defenses [40]. A pregnancy being carried to term therefore depends on processes that suppress maternal immunity directed against fetal alloantigens. Maternal exposure to the seminal fluid and the timing of sexual intercourse between the father and the mother before the initiation of pregnancy may have an important role. The deposition of seminal fluid in the female genital tract, in fact, has the ability to initiate, as early as ejaculation, an immune response, predominantly T helper-2 type, to the disadvantage of T helper-1, which instead is associated with insufficient placentation [41]. In another pathway, maternal immune tolerance to fetal HLA-A and HLA-B antigens on the paternal side results from a sort of immunological memory developed during previous exposures to the same antigens contained in seminal fluid. The inverse relationship between the duration of sexual cohabitation and the incidence of pre-eclampsia may make sense in terms of evolution. In fact, human women are among the few mammals who conceive (generally) after multiple exposures to seminal fluid; this might make sense in the evolution of the species considering that those born to a stable couple would be better conceived and better cared for subsequently. Pre-eclampsia, moreover, is a complication typical of humans compared to all other mammals. Considering that the major difference in the human embryo lies in the size of the brain, which requires about 60% of the nutrients during the extraordinary phase of development in the second and third trimesters, and thus, the optimal invasion of the trophoblast into maternal tissues, the immunological compromise between the mother and father surely must be superior. According to Robillard’s theory [42], the low fertility rate of the human female (about 25%) is the price the human species pays for having a brain of large size, which, in fact, requires good trophoblastic invasion, with a relatively low rate of pre-eclampsia. The average wait of 7–8 months before conception by a newly established couple represents the mechanism by which the human female protects herself from pre-eclampsia by preserving the possibility of reproducing again, perhaps with the same partner [43]. Data from assisted reproductive technology seem to confirm similar associations: pregnancies achieved through a sperm donor have a significantly increased risk of developing pre-eclampsia [44], while pregnancies after double gamete donation are at about a 3 times higher risk of developing pre-eclampsia compared with oocyte donation alone [45] and with standard in vitro fertilization [46].

## 5. Father–Mother Competition

**Figure 1:**
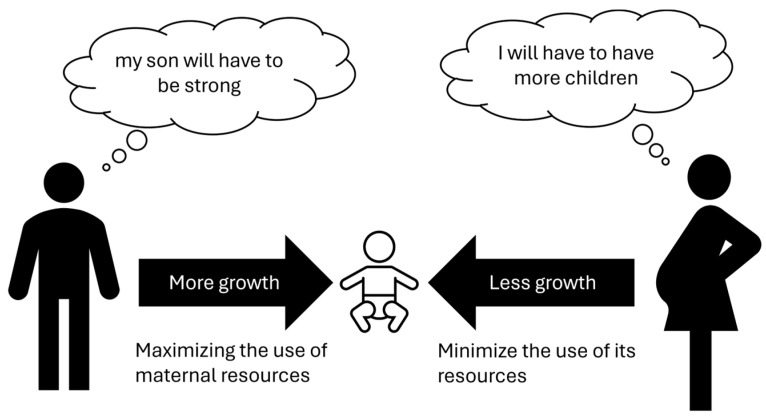
Maternal–paternal conflict theory.

Modern genetics indicates that for specific genes only one of the two alleles is active, in some cases only the maternal one, in others only the paternal one. This phenomenon is called “genomic imprinting” and explains some diseases that do not follow Mendelian heredity. Experiments in the reproductive field have clarified some aspects of such hereditary mechanisms. Laboratories modified oocytes to create two kinds of embryos: “gynogenetic” with dual maternal genetic heritage and “androgenetic” with only dual paternal heritage. The first type died within a few days due to the lack of extraembryonic tissues (trophoblast); the second one reached the stage of blastocyst but not implantation [47], suggesting a main role for paternal genes. Interestingly, similar phenomena occur in teratoma (or dermoid cysts). In this type of pathological tissue, only duplicate maternal genetic material is present that can generate embryonic tissues derived from all three germ leaves (ectoderm, endoderm, and mesoderm). In fact, in these cysts, hair, skin with its adnexa, glands, and nerves may be present [48]. In contrast, the complete hydatidiform molar contains exclusively duplicate paternal genetic inheritance and is not capable of generating an embryo, only an extraembryonic trophoblast [49]. These data suggest that paternal genes have a pivotal role in promoting placental growth, while maternal genes have a main role in the formation of the embryo. Proper embryo–fetal development requires both maternal and paternal components as well as the selective inactivation of an allele (genomic imprinting), which is a complex phenomenon that must be well regulated for optimal embryo–fetal growth. An interesting theory concerning this matter was postulated as a kind of “maternal-paternal conflict” [47,48,49,50]. Although the common objective of the male and the female is the survival of the species, this should take place as much as possible in polygamous conditions so that as many genes as possible are inherited. Such a view leads to conflict, in that the father’s genes are interested in the greatest possible growth of the fetus and, therefore, of the future infant, through the development of the placental tissue; on the other hand, the mother would be more interested in not spending too many resources for each individual child, so that she can be immediately ready for conception with another male, so she would tend to genetically “slow down” fetal growth. Into this conflicting game would be inserted an imprinting genomic that would regulate fetal growth by drawing on genetic resources in a modulated manner from both parents (Figure 1). It is interesting, from this perspective, to consider the difference between humans and birds, where the amount of nutrition for the embryo is pre-determined in the egg and cannot undergo subsequent modulations.

## 6. The Father, the Fetus, and the Future Offspring

The paternal origins of health and disease (POHaD) concept can be evidenced from the early stages of fetal life because of the influence of genetic and environmental epigenetic modifications of spermatozoa and seminal plasma on fetoplacental development. In the last few decades, several studies have highlighted that paternal age, nutrition, lifestyle, environmental/occupational exposure, body mass index (BMI), and diabetes may have an impact on fetal health and future offspring.

### 6.1. Paternal Age

Even in the absence of an accepted definition of advanced paternal age (APA) in the current literature, the father’s aging leads to several modifications of sperm cells: decreased sperm quality [51], increased DNA damage [52,53], telomere elongation [54,55,56], centrosome aberrations [57], de novo DNA mutations [58], aneuploidies [59], and epigenetics alterations [51]. These sperm modifications harm fertility, decrease pregnancy rates in assisted reproductive technology, and increase the risk of miscarriage [51], aneuploidies [59], congenital anomalies (cleft palate, limb defects, musculoskeletal anomalies, patent ductus arteriosus) and rare syndromes related to fibroblast growth factor receptor (FGFR) mutations (achondroplasia, thanatophoric dysplasia, osteogenesis imperfecta, Apert syndrome, Pfeiffer syndrome, Crouzon syndrome). Neurofibromatosis and Marfan syndrome have also been consistently reported more frequently in the offspring of older fathers [51,60]. Moreover, compared to younger fathers of 25–29 years, paternal age over 45 years increases the risk of low birth weight (LBW) [61], PTB [62,63], and late stillbirth [64,65,66]. APA also adversely affects the offspring, maybe through an accumulation of de novo mutations in sperm cells [51,67,68,69]. It has been shown that children with older fathers are at higher risk for pediatric malignancies (acute lymphoblastic leukemia, retinoblastoma, breast and central nervous system cancers) [51,58,70,71,72] and neuropsychiatric disorders (bipolarism, autism, schizophrenia) [61,73,74,75]. Parental seniority can also adversely affect the future of offspring, as advanced age increases the likelihood of illness that would force children into early caregiving and increases the likelihood of death that would compromise their economic status and happiness [76]. The problem of parental seniority also occurs in the context of de novo genetic mutation transmission. Monogenic disorders (MDs) that share a combination of epidemiological and molecular features have been grouped into a distinct class of MDs with a high rate of de novo cases, termed paternal age effect disorders (PAEs). In these cases, there is an increased rate of germline mutations, paternal origin of the mutations, advanced paternal age, and involvement in the rat sarcoma virus protein–mitogen-activated protein kinase (RAS-MAPK) pathway. The risk of an aging father having a child with a de novo PAE disorder is at least 1 in 200 [77]. Infants with PAE develop congenital skeletal defects, including facial dysmorphia, craniosynostosis, syndactyly, short stature, hypertelorism, or marfanoid habitus. In addition, some diseases are associated with cardiac malformations, neurological defects, or predisposition to cancer [78]. Pathogenic variants of the FGFR2 gene can lead to syndromes such as Crouzon (OMIM 612247), Pfeiffer (OMIM 612247), and Apert (OMIM 123500). MD PAEs include multiple endocrine neoplasia type 2A (OMIM 171400) and 2B (OMIM 162300), i.e., marfanoid habitus, gastrointestinal tract defects, and increased risk of developing bone marrow and thyroid cancer [79]. Similarly, patients with Noonan and Costello (OMIM 218040) syndromes, caused by the PTPN11 and HRAS genes, have higher risk of childhood cancers, facial dysmorphia, cardiac disorders, neurocognitive delays, and musculoskeletal and cardiac abnormalities [80] (Table 1).

### 6.2. Paternal Nutrition

In animal models, there is growing evidence that the nutritional status of the father, as with that of the mother, strongly influences the health of the fetus and its progeny. How this can happen is still little known, but seems to be based on epigenetic mechanisms (DNA methylation, histone modifications) and/or small non-coding alterations of spermatic RNA (sncRNAs) [81]. In rodents, paternal folate deficiency during the periconceptional period is linked to a higher rate of post-implantation embryo loss, fused and abnormal placentas, fetal abnormalities (craniofacial and limb defects), delayed muscle/skeletal development, and an increased vulnerability to anxiety and depression in the offspring [82,83]. In chickens, folic acid supplementation could also impact spermatozoa mRNA expression and induce transgenerational metabolic changes [84,85]. Fathers on a low-protein diet will have pups who are more likely to develop glucose intolerance, metabolic and cardiovascular dysfunction, an impaired skeletal system, and impaired bone deposition [86,87,88,89] with an increased risk of breast cancer in a mouse model [90]. Moreover, the up-regulation of genes coding for nutrient transporters in the placenta and of genes involved in fetal growth regulation is also reported [89,91]. Paternal food restriction seems to impair paternal fertility and increase dyslipidemia and adiposity prevalence in the offspring [91]. Also, a high-fat diet (HFD) negatively affects embryo development and the health of future progeny in rodents. Paternal HFD exposure leads to a reduced rate of ongoing pregnancy, decreased fetal and placental weights, delayed limb morphology, a decreased crown-rump length, and an increased risk in the offspring of chronic kidney diseases and metabolic syndrome-like characteristics; most recently, this has been found to occur through the transmission of β-cell dysfunction and glucose intolerance in F1 female offspring and altered insulin sensibility in F1 male offspring [83,92,93]. HFD also impairs hippocampal neurogenesis, maybe through increased methylation of the brain-derived neurotrophic factor (BDNF) gene promoter on spermatozoa, with resulting cognitive impairment in the F1 generation [94]. In the end, a paternal high-sugar diet seems to induce the up-regulation of some specific subtypes of tRNA-derived small RNAs (tsRNAs) of sperm cells that alter energy homeostasis in the offspring [81,95].

### 6.3. Paternal Lifestyle

Paternal “unhealthy habits” during the pre-conception period can profoundly impair the father’s sexual organs, the fetus, and the offspring’s health. Tobacco smoke contains several toxic and mutagenic substances that have a deep impact not only on male fertility but also on the genetics of the non-smoking descendant. The chemicals of cigarettes act as endocrine disruptors and increase the formation of reactive oxygen species (ROS) and secretion of inflammatory cytokines, leading to the development of oxidative stress (OS), DNA damage, and germ cell apoptosis. In addition, tobacco smoke is a mutagen and an aneugen of spermatozoa, where the genetic mutations can be transferred to the progeny [96]. This is why there is evidence that paternal pre-conception cigarette smoking increases the risk of adverse birth outcomes, including spontaneous abortion, preterm birth, aneuploidy, heart malformations (conotruncal defects, septal and left ventricular outflow tract obstructions defects), clefts, anorectal malformations, and childhood leukemia (acute myeloid leukemia/acute myeloblastic leukemia) [97,98,99,100,101]. Moreover, a father smoking before the age of 15 seems to be associated with an increased risk of asthma in his progeny [102]. As for smoking, paternal excessive alcohol intake deteriorates sperm quality and induces epigenetic changes, as well as DNA damage, in the testicular germline and sperm [103]. However, the effects on the fetus and offspring need to be further investigated, as there are different and inconsistent findings in the literature. Some data underline a possible association between paternal smoking and alcohol intake during the prenatal period and impaired offspring mental health (especially hyperactivity/Attention Deficit Hyperactivity Disorder (ADHD)) [104]. Recreational drugs, as with other ʺunhealthy habitsʺ, impair seminal parameters. Paternal cocaine and opioid addiction seems to be associated with LBW, premature deaths before the age of 6, and severe forms of ADHD in the offspring [105,106,107]. Finally, the role of paternal physical activity and caffeine use in the pre-conception period is almost unexplored in the current literature. However, regular exercise in male mice has shown increased insulin sensitivity in their offspring, due to the different methylation status of glucose metabolism genes of paternal sperm and skeletal muscle [108].

### 6.4. Paternal Environmental/Occupational Exposure

In general, men are more frequently exposed to harmful substances in the workplace and during everyday life than women, especially because of their lack of awareness of the potential reproductive risks. Research concerning mechanisms regarding the impact of environmental and occupational paternal exposure on pregnancy and offspring is growing. There are many professional agents, potentially teratogenic, that, to different degrees and with varying durations of exposure, can induce functional, epigenetic, and genetic changes in sperm cells. These may include chemicals and radiation. However, the current literature is still limited on this specific topic; in fact, most studies concern parental exposures and not just paternal exposures. Occupational exposure, even of only one parent, to organic solvents, phthalates, alkylphenolic compounds, herbicides, and pesticides is associated, respectively, with an increased risk of anencephaly [109], peri-membranous ventricular septal defects and pulmonary valve stenosis, ventricular septal defects [110], astrocytoma [111], and astroglia brain tumors [112]. In fact, there is an increased risk of fetal death from congenital anomalies for the offspring of agricultural workers, due to pesticide exposure [113]. Children of painters and carpenters regularly exposed to solvents are at greater risk of congenital abnormalities (anencephaly, heart defects, neural tube defects) [114,115]. In addition, exposure to ionizing agents appears to be a risk factor for PTB and LBW [116]. Regarding environmental exposure, several toxic agents have been identified in recent decades as endocrine disruptors (CDEs).

TCDD (2,3,7,8-tetrachlorodibenzo-p-dioxin) is an example of EDCs, and its paternal exposure in pups is found to be related to IUGR due to placental dysfunction and alterations in the expression of Insulin-like Growth Factor 2 (IGF2) through epigenetic changes in the male germ cell line in a mouse model [117,118]. Bisphenol-A (BPA) is one of the most used ECDs, with widespread application in the manufacture of plastics and epoxy resins. There is evidence that paternal exposure to BPA induces physiological and functional disruption in male germ cells that impairs the reproductive health of the progeny in mice [116]. Regarding air pollutants, a recent study demonstrates that paternal exposure to PM ₂₅ programs the energy homeostasis of the offspring [119].

### 6.5. Paternal Body Mass Index

There is increasing awareness that paternal weight before conception can affect not only sperm quality but also genomic imprinting in germ cells, with a great impact on the health of the fetus, the placenta, and the offspring. Recent cohort studies demonstrate that paternal obesity is associated with an increased risk of hypertensive disorders of pregnancy, macrosomia, PTB, and small for gestational age (SGA) [120,121,122]. In addition, studies have demonstrated a higher incidence of asthma; hand, foot, and mouth disease; anemia; dental caries; and obesity in adolescents of obese fathers [120,123,124]. The mechanism underlining these transmissions is still almost unexplored. There is evidence in mice that paternal obesity induces placental hypoxia and sex-specific impairments in placental vascularization and offspring metabolism [125]. Paternal obesity in mice has shown increased endoplasmic reticulum (ER) stress-related protein levels in the fetal liver and altered hepatic expression of gluconeogenic factors at E18.5. These prenatal modifications result in glucose intolerance and impaired energy metabolism in the offspring. In humans, researchers have shown a significant decrease in methylation among newborns of obese fathers in the IGF2 methylated regions (DMRs) in DNA extracted from cord blood leucocytes [126]; hypomethylation at IGF2 DMR is associated with higher circulating IGF2 levels in the offspring [127,128].

### 6.6. Paternal Diabetes

The complex etiology and the exponential increase in type 2 diabetes (T2DM) highlight the need to find alternative strategies able to combat this public health concern. It is well established in literature that T2DM can be programmed from the early stages of life when there is an adverse intrauterine environment. What still needs to be clarified is whether there is a role of the father in the programming of T2DM, because this specific topic is still almost not investigated. Fornes et al. have shown that paternal T2DM affects fetal overgrowth, placental development, and sex-dependent lipid metabolism in rats. Specifically, they observed that only the placentas of male fetuses from diabetic fathers showed an increase in lipid reserve and in the mRNA expression of enzymes involved in lipid oxidation and transport pathways [129].

## 7. Conclusions

This review has covered all the major aspects of the father’s role in determining the onset of pregnancy, its possible evolution, major obstetrical diseases, and neonatal outcomes. Studies show that the father possesses genetic and environmental responsibility in determining pregnancy outcomes. Most of the literature focuses on maternal factors that may determine the initiation, course, and outcome of pregnancy. Paternal factors, less studied and less considered, may influence in the possibility of fertilization through the quality and genetics of spermatozoa. The father’s lifestyle also impacts these factors. In the early stages of pregnancy, paternal genetics affects fetal development, since genes inherited from the father work on the fetal side of the intervillous space. Immunological aspects seem to intervene, especially in the multifactorial genesis of preterm delivery and the development of pre-eclampsia. With this in mind, some authors have also hypothesized a “mother-father conflict” from a species evolution perspective. However, in addition to biological aspects, environmental and socio-cultural aspects should not be overlooked. A father’s lifestyle probably also influences the outcome of pregnancy by determining the environment in which the mother lives. Responsibility is determined not only as an “involuntary” factor, that is, in transferring his genes, but also as a “voluntary” and “behavioral” factor, since the lifestyle, diet, and physical well-being with which a father presents himself at the appointment with procreation can affect the existence and well-being of an infant. From an epigenetic perspective, this responsibility becomes transgenerational in that the negative aspects of a father are passed on to a child, who may then become a father in turn. All these concepts are extremely useful for personalized obstetrical care and in the field of medically assisted procreation where gamete selection can take place the future considering the characteristics of both the mother and father.

## Figures and Tables

**Table 1:** Paternal age effect (PAE) disorders.

Disorders
Achondroplasia
Apert syndrome
Cardiofaciocutaneous syndrome
Costello syndrome
Crouzon syndrome
Hypochondroplasia
Muenke syndrome
Multiple endocrine neoplasia types 2A, 2B
Noonan syndrome
Pfeiffer syndrome
Thanatophoric dysplasia

## Data Availability

Not applicable.
